# FOCUSING ON DIVERSITY: UTMB RECRUITMENT STRATEGY FOR THE D-CARE STUDY

**DOI:** 10.1093/geroni/igac059.1209

**Published:** 2022-12-20

**Authors:** Rafael Samper-Ternent, Alice Williams, Roxana Hirst, Rebecca Galloway, Elena Volpi

**Affiliations:** University of Texas Medical Branch, Galveston, Texas, United States; Sealy Center on Aging, University Of Texas Medical Branch, Galveston, Texas, United States; Sealy Center on Aging, University Of Texas Medical Branch, Galveston, Texas, United States; Sealy Center on Aging, University Of Texas Medical Branch, Galveston, Texas, United States; University of Texas Medical Branch, Galveston, Texas, United States

## Abstract

The University of Texas Medical Branch (UTMB) is the main healthcare system in Galveston County where about 15% of older adults identify as Hispanic. Our recruitment efforts for the Dementia (D-CARE) study included adapting and translating in Spanish the screening, recruitment, and intervention materials. The study is being conducted by a bilingual team, and actively engages a highly diverse Local Patient and Stakeholder Council. After obtaining a partial HIPAA waiver from the Institutional Review Board, we created a dementia registry in the UTMB Epic (TM) electronic health record which captured patients diagnosed with ICD-9/10 codes of dementia. Referral letters from UTMB primary care and neurology providers authorized us to contact eligible patients. We utilized outpatient clinic appointment schedules, direct referrals, and community outreach to meet our enrollment goal. This recruitment strategy resulted in inclusion of 478 patient-caregiver dyads with 27.4% of participants identifying as Hispanic and 17% as Black.

